# Effect of diet‐induced weight loss on angiopoietin‐like protein 4 and adipose tissue lipid metabolism in overweight and obese humans

**DOI:** 10.14814/phy2.13735

**Published:** 2018-07-12

**Authors:** Birgitta W. van der Kolk, Roel G. Vink, Johan W. E. Jocken, Nadia J. T. Roumans, Gijs H. Goossens, Edwin C. M. Mariman, Marleen A. van Baak, Ellen E. Blaak

**Affiliations:** ^1^ Department of Human Biology NUTRIM School of Nutrition and Translational Research in Metabolism Maastricht University Medical Center^+^ Maastricht the Netherlands

**Keywords:** Adipose tissue, angiopoietin‐like protein 4, lipid metabolism, lipoprotein lipase, weight loss

## Abstract

Angiopoietin‐like protein 4 (ANGPTL4) plays a role in lipid partitioning by inhibiting lipoprotein lipase (LPL)‐dependent plasma clearance of triacylglycerol in adipose tissue. We investigated the effects of diet‐induced weight loss on plasma ANGPTL4 concentrations in relation to in vivo adipose tissue LPL activity and lipolysis and adipose tissue ANGPTL4 release in overweight/obese participants. Sixteen individuals (BMI: 28–35 kg/m^2^; 10 women) were randomized to a dietary intervention composed of either a low‐calorie diet (1250 kcal/day) for 12 weeks (*n* = 9) or a very low‐calorie diet (500 kcal/day) for 5 weeks, followed by a 4‐week weight stable period. Before and after the intervention, we measured arteriovenous concentration differences in combination with adipose tissue blood flow before and after intake of a high‐fat mixed meal with [U‐^13^C]‐palmitate to assess in vivo adipose tissue LPL activity and lipolysis. The intervention significantly reduced body weight (−8.6 ± 0.6 kg, *P *<* *0.001). Plasma ANGPTL4 concentrations were unaffected. Significant postprandial adipose tissue ANGPTL4 release into the circulation was observed (*P *<* *0.01). No association was observed between plasma ANGPTL4 and in vivo LPL activity. After intervention, fasting and postprandial plasma ANGPTL4 concentrations were positively associated with adipose tissue nonesterified FA (NEFA) and glycerol release, reflecting in vivo adipose tissue lipolysis (fasting NEFA:* P *=* *0.039 and postprandial NEFA:* P *=* *0.003). In conclusion, plasma ANGPTL4 is unaffected by weight loss and is secreted from human adipose tissue after a high‐fat meal in overweight/obese participants. Plasma ANGPTL4 concentrations were not related to in vivo adipose tissue LPL activity, but were positively associated with in vivo adipose tissue lipolysis after weight loss.

## Introduction

Multiple physiological and environmental factors are known to interact in the regulation of energy intake and fuel metabolism to maintain energy homeostasis in humans. Among them, the enzyme lipoprotein lipase (LPL) plays an important role in fasting and postprandial lipid fuel partitioning in humans (Kersten 2014). LPL is located at the capillary endothelium where it catalyzes the hydrolysis of triacylglycerol (TAG)‐rich plasma lipoproteins to glycerol and nonesterified fatty acids (NEFAs) (Goldberg et al. 2009). The resulting NEFAs are then taken up by the peripheral tissues via passive diffusion or specialized transport proteins (i.e., FAT/CD36) and will be directed toward oxidation or storage, or spilled over into the circulation (Miles et al. 2004; Bickerton et al. 2007).

The activity of LPL is under tight tissue‐specific nutritional and hormonal control. For instance, insulin is a major activator of adipose tissue LPL activity which results in postprandial (chylomicron)‐TAG storage (Kersten 2014), while in skeletal muscle insulin suppresses LPL activity in healthy humans (Farese et al. 1991). In obesity, a diminished lipid‐buffering capacity of the adipose tissue might lead to ectopic lipid deposition in skeletal muscle, liver, and heart and has been linked to insulin resistance in these tissues (Stinkens et al. 2015). Next to insulin, angiopoietin‐like protein 4 (ANGPTL4) has emerged as an important regulator of in vivo LPL activity in adipose tissue and skeletal muscle, and thereby as a potent modulator of lipid metabolism (Dijk and Kersten 2014).

ANGPTL4 is a central factor of a feedback mechanism that regulates plasma TAG hydrolysis during various physiological conditions (Dijk and Kersten 2016). ANGPTL4 is expressed and secreted by numerous cell types, including human adipocytes. Tissue‐specific expression of ANGPTL4 is induced by fatty acids (FAs) via peroxisome proliferator‐activated receptors (PPARs) in response to changes in lipid availability and cellular fuel demand (Dijk and Kersten 2016). It has been shown that during long‐term fasting (Kersten et al. 2009) and following acute physical activity (Kersten et al. 2009; Catoire et al. 2014; Norheim et al. 2014) skeletal muscle ANGPTL4 expression and plasma ANGPTL4 concentrations are increased in humans.

In rodent adipose tissue studies, under fasting conditions, increased ANGPTL4 concentrations inhibit adipose tissue LPL activity and might therefore lead to inhibited plasma TAG clearance by adipose tissue, while this effect was blunted in ANGPTL4 knockout mice (Kroupa et al. 2012). Moreover, it has been shown that ANGPTL4 promotes intracellular degradation of LPL (Dijk et al. 2016) and intracellular lipolysis (Gray et al. 2012) in murine cell studies. Subsequently, ANGPTL4 is hypothesized to play a role in lipid partitioning to other tissues (i.e., muscle and liver) under conditions with increased energy demand, like fasting and exercise (Dijk and Kersten 2016). However, up to now data on circulating ANGPTL4 in relation to in vivo adipose tissue FA metabolism in humans are missing.

In addition, no information is available on the role of ANGPTL4 in the impaired adipose tissue TAG extraction as observed under obese conditions. After short‐term and chronic caloric restriction, circulating ANGPTL4 concentrations were increased (Kersten et al. 2009; Jonker et al. 2013), indicating a role in fuel partitioning also in humans. Importantly, in these studies, participants were in a negative energy balance when plasma ANGPTL4 was measured. Therefore, the distinction between acute energy restriction and the effect of weight loss per se on plasma ANGPTL4 cannot be made.

The origin of plasma ANGPTL4 is currently unknown. Based on human ANGPTL4 mRNA expression and ANGPTL4 overexpression studies in mice, it has been postulated that the liver is the main contributor to systemic ANGPTL4 concentrations (Köster et al. 2005; Kersten et al. 2009). Intestine and skeletal muscle might also contribute to circulating ANGPTL4 concentrations (Alex et al. 2013; van der Kolk et al. 2016a). Nevertheless, ANGPTL4 was originally discovered as fasting‐induced adipose factor (FIAF), which suggests that adipose tissue might also be an important contributor to circulating ANGPTL4 concentrations (Kersten et al. 2000). However, data on the in vivo contribution of adipose tissue to circulating ANGPTL4 levels in humans is still missing.

The aim of the present study was to investigate fasting and postprandial plasma ANGPTL4 concentrations and their relationship with in vivo adipose tissue LPL activity and adipose tissue lipolysis in overweight and obese humans before and after diet‐induced weight loss. Secondly, we examined whether human adipose tissue secretes ANGPTL4 into the circulation under fasting conditions and after the intake of a high‐fat mixed‐meal and if this is affected by significant weight loss. In addition, adipose tissue biopsies were taken to assess transcriptional changes in FA metabolism before and after weight loss.

## Materials and Methods

### Study participants

This study is part of a larger study in which 57 individuals with overweight and obesity (BMI 28–35 kg/m^2^) were recruited by advertisement via local media (Vink et al. 2016). Sixteen individuals with overweight and obesity (BMI 28–35 kg/m^2^), 10 women and 6 men were included in the present study, as reported previously (Vink et al. 2017a). All results are shown for this subgroup. Overall, these individuals had no metabolic disturbances and had to remain weight stable (weight change <3.0 kg) for 2 months prior to the start of the study. All participants gave their written informed consent before participation in the study. The study was performed according to the declaration of Helsinki and was approved by the Medical Ethics Committee of Maastricht University Medical Centre.

### Study design

As described previously (Vink et al. 2016), participants followed a dietary intervention program that was divided in three periods: a 12‐week low‐calorie diet (LCD‐period) or 5‐week very low‐calorie diet (VLCD) weight loss period (WL), a 4‐week weight stable period (WS), and a 9‐month follow‐up period. The WL period and WS period taken together was named the dietary intervention (DI) period. Fasting and postprandial FA metabolism and ANGPTL4 measurements were studied before and after the DI‐period. Participants were randomly assigned to either the LCD (*n* = 9) or VLCD (*n* = 7) group and both interventions aimed at a weight loss of approximately 10%. In the 12‐week program participants followed a LCD providing 1250 kcal/day designed by the dietician. The participants could consume one meal that was replaced by meal replacements (Modifast; Nutrition et Santé Benelux, Breda, The Netherlands), two meals that were prepared by the participants themselves and three in‐between meal snacks. In the VLCD group participants followed a 5‐week diet in which three meals per day were replaced by meal replacements, providing 500 kcal/day. During this period, participants could consume an unrestricted amount of low‐calorie vegetables. The detailed composition of the diets has been described previously (Vink et al. 2016). Both groups subsequently underwent a 4‐week WS period with a diet based on the energy requirements of the participants. This allowed us to investigate the effect of weight loss, without the interfering effect of a negative energy balance. The study dietician provided dietary advice to both groups to assist with losing weight during the WL period (five meetings) and in remaining weight stable throughout the WS period (four meetings). The researchers, study participants, and dietician were not blinded to the intervention. This trial is registered with http://www.clinicaltrials.gov: as NCT01559415.

### High‐fat mixed‐meal test

Participants were studied after an overnight fast and were asked to refrain from strenuous exercise and drinking alcohol 24 h before the study day. In addition, they were asked to avoid consuming food products naturally enriched with [^13^C] for 7 days before the study day. Adipose tissue metabolism was studied using arteriovenous concentration differences combined with measurements of adipose tissue blood flow (ATBF). Two catheters were inserted before the start of the experiment. One catheter was placed retrogradely into a superficial dorsal hand vein, which was heated in a hot‐box (60°C) to obtain arterialized blood. To sample blood from the venous effluent of subcutaneous abdominal adipose tissue, another catheter was placed in a superficial epigastric vein (Jocken et al. 2008). This catheter was kept patent by continuous saline infusion. Blood samples were obtained from both sites simultaneously. Blood samples were taken at two time points during fasting: t‐30 min and just before meal ingestion (t0). Postprandial blood samples were taken at five time points (t60, t120, t180, t240, t300 min) after consumption of a high‐saturated FA (SFA) mixed meal (t0) containing 200 mg [U‐^13^C]‐palmitate (98% enrichment, Cambridge Isotope Laboratories). The liquid meal provided 2.6 MJ, consisting of 61 Energy % (E%) fat (35.5 E% SFA, 18.8 E% MUFA, and 1.7 E% PUFA), 33 E% carbohydrates and 6.3 E% protein. The participants were asked to drink the milkshake within 10 min.

Abdominal subcutaneous ATBF was measured on the contralateral side of the abdomen, at the same level where the epigastric vein was cannulated. Fasting and postprandial ATBF was determined using the ^133^Xe washout technique (Jocken et al. 2008).

### Plasma ANGPTL4 and metabolite concentrations

ANGPTL4 was measured in EDTA plasma before and after the DI‐period during the high‐fat mixed‐meal test by ELISA, as described previously (van der Kolk et al. 2016a). Briefly, 96well plates were coated with antihuman ANGPTL4 polyclonal goat IgG antibody (AF3485, R&D Systems) and incubated overnight at 4°C. Plates were washed extensively between each step. After blocking, 100 *μ*L of 20‐fold diluted human plasma was added, followed by 2 h incubation at room temperature. A standard curve was prepared using recombinant ANGPTL4 (3485‐AN, R&D Systems) at 0.3–2.1 ng/well. Next, 100 *μ*L of diluted biotinylated antihuman ANGPTL4 polyclonal goat IgG antibody (BAF3485, R&D Systems) was added for 2 h, followed by addition of streptavidin‐conjugated horseradish peroxidase for 20 min and tetramethyl benzidine substrate for 6 min. The reaction was stopped by addition of 50 *μ*L of 10% H_2_SO_4_, and the absorbance was measured at 450 nm. Details on other biochemical analyses and hematocrit have previously been reported (Vink et al. 2017a).

### Adipose tissue biopsy

Before and after the DI‐period abdominal subcutaneous adipose tissue needle biopsies (~1 g) were collected 6–8 cm lateral from the umbilicus under local anesthesia (2% lidocaine) by needle biopsy. Biopsies were immediately rinsed with sterile saline and visible blood vessels were removed with sterile tweezers. Adipose tissue biopsies were snap‐frozen in liquid nitrogen and stored at −80°C for further analysis.

### Adipose tissue microarray analyses

RNA was extracted from frozen adipose tissue (~150 mg) using Trizol chloroform extraction (Invitrogen, Breda, The Netherlands). Next, total RNA (100 ng per sample) was labeled by Whole‐Transcript Sense Target Assay and hybridized to human whole‐genome Affymetrix Gene 1.1 ST arrays targeting 19,654 unique genes (Affymetrix, Santa Clara, CA). Quality control and data analysis have been described in detail previously (Vink et al. 2017a). We performed a supervised gene expression analysis focusing on 22 genes important for uptake, storage, and release of FA by adipose tissue (Table S1) and analyzed gene expression before and after DI‐period. Changes in mRNA levels were defined as significantly different when the *q*‐value was <0.05 in a paired *t*‐test with Bayesian correction (Limma). Array data have been submitted to the Gene Expression Omnibus (number GSE77962).

### Calculations

Plasma tracer concentrations were calculated by multiplying the TTRs of [U‐^13^C] palmitate with the corresponding palmitate–NEFA or palmitate–TAG concentrations. To calculate the net fluxes of metabolites across subcutaneous abdominal adipose tissue, the arteriovenous concentration difference was multiplied by adipose tissue plasma flow. Plasma flow was calculated by multiplying ATBF with: 1 – (hematocrit/100). A positive flux indicates net uptake across adipose tissue, whereas a negative flux indicates net release. In vivo LPL activity was based on net fluxes of [U‐^13^C]‐labeled palmitate (Bickerton et al. 2007; van der Kolk et al. 2016b). Postprandial areas under the curve (AUC) of metabolites were calculated using the trapezium rule.

### Statistics

Data are presented as mean ± SEM. Comparisons of variables between time‐points within groups were made with the Students’ paired‐sample *t*‐test. Between‐group comparisons were made with the independent samples *t*‐test. Variables were evaluated for residual normality and logarithmic transformations were performed when appropriate. For non‐normally distributed variables the logarithmic transformed *P*‐values are shown, whereas values of concentrations are presented as nonadjusted. Statistical calculations were performed with SPSS for Macintosh, Version 22 (Chicago, IL). *P *<* *0.05 was considered statistically significant.

## Results

### Study population

Participant characteristics are summarized in Table 1. The present study included participants that lost weight either by a LCD (*n* = 9) or VLCD diet (*n* = 7). Similar weight loss was achieved by both diets (LCD: −8.6 ± 0.9 kg and VLCD: −8.7 ± 0.9 kg; *P *=* *0.927) and no significant differences between diet groups were observed for the FA metabolism parameters (data not shown). Therefore we analyzed both diet groups as a single group (Vink et al. 2016). Significant improvements in body fat percentage, waist‐hip ratio, and fat cell size were observed, as reported previously (Vink et al. 2017a) (Table 1).

**Table 1:** Characteristics of participants

	Study start	End of DI‐period	*P*‐value
Diet (LCD/VLCD)	9/7		
Sex (female/male)	10/6		
Age (year)	48.8 ± 1.8		
Weight (kg)	96.0 ± 2.4	87.3 ± 2.1	<0.001
BMI (kg/m^2^)	32.4 ± 0.6	29.6 ± 0.6	<0.001
Waist circumference (cm)	103.3 ± 2.5	96.0 ± 1.8	<0.001
Fat mass (%)	43.2 ± 2.3	38.1 ± 2.8	<0.001
Systolic blood pressure (mmHg)	123.7 ± 3.0	118.9 ± 2.8	<0.001
Diastolic blood pressure (mmHg)	84.9 ± 1.8	79.2 ± 1.8	<0.001
Glucose (mmol/L)	5.3 ± 0.1	5.2 ± 0.1	0.005
Insulin (mU/L)	16.3 ± 1.1	13.8 ± 1.5	0.248
HOMA‐IR	3.8 ± 0.4	3.2 ± 0.4	0.113
NEFA (*μ*mol/L)	460 ± 51	521 ± 70	0.440
TAG (*μ*mol/L)	1375 ± 143	972 ± 109	0.001
Total cholesterol (mmol/L)	6.3 ± 0.2	5.9 ± 0.2	0.017
Adipocyte size (*μ*m)	68.9 ± 1.2	64.7 ± 1.3	0.005

Values are mean ± SEM. *P*‐value for difference between start study and after DI‐period, student's *t*‐test for paired samples.

### Weight loss does not alter ANGPTL4 response to high‐fat meal

**Figure 1:**
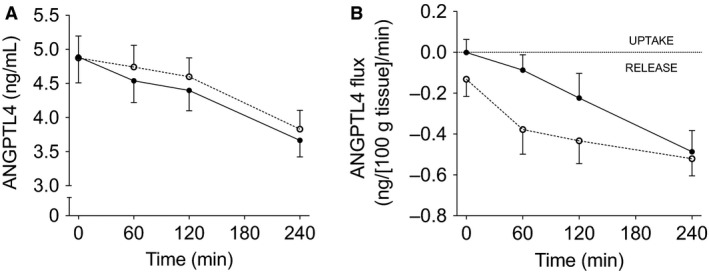
Arterialized plasma ANGPTL4 concentrations (A) and net ANGPTL4 flux across adipose tissue (B) under fasting conditions (*t* = 0) and after consumption of a high‐SFA meal. Black symbols; baseline measurements, White symbols; after DI‐period. Values are mean ± SEM.

We investigated fasting and postprandial plasma ANGPTL4 concentrations following consumption of a high‐SFA meal. Arterialized ANGPTL4 concentrations during fasting and the postprandial decrease in arterialized ANGPTL4 concentrations were comparable before and after DI‐period (Fig. 1A). This indicates that weight loss *per se* does not alter fasting and postprandial plasma ANGPTL4 responses.

### ANGPTL4 is released from adipose tissue

Next, it has been postulated that adipose tissue may be a source of plasma ANGPTL4 in humans (Dijk and Kersten 2014). In the present study, no significant release of ANGPTL4 from adipose tissue during fasting conditions was observed before and after the DI‐period (Fig. 1B). However, significant postprandial ANGPTL4 release from adipose tissue was detectable following a high‐SFA meal before (AUC_0–4 h_; −0.23 ± 0.08 ng∙100 g tissue^−1^∙min^−1^ vs. zero flux; *P *=* *0.011) and after the DI‐period (AUC_0–4 h_; −0.39 ± 0.07 ng∙100 g tissue^−1^∙min^−1^ vs. zero flux; *P *<* *0.001) (Fig. 1B). After the DI‐period, this postprandial ANGPTL4 release tended to be higher when compared with before weight loss (*P *=* *0.072).

### Systemic responses and adipose tissue FA metabolism after a high‐SFA meal

**Figure 2:**
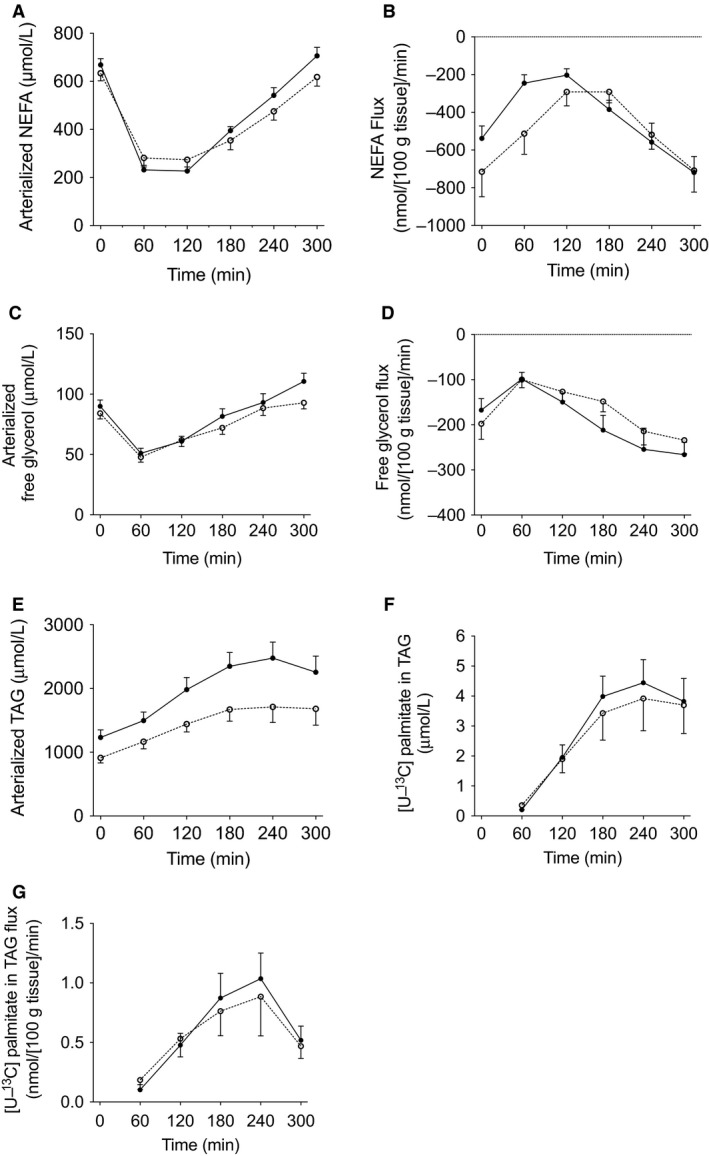
Postprandial lipid metabolism. Arterialized plasma NEFA concentrations (A), the net flux of NEFA across adipose tissue (B), arterialized plasma glycerol concentrations (C), the net flux of glycerol across adipose tissue (D), arterialized plasma TAG concentrations (E), [U‐^13^C]‐palmitate concentrations in the plasma TAG fraction (F), and the net flux of [U‐^13^C]‐palmitate TAG (G) across adipose tissue during fasting conditions (*t* = 0) and after consumption of a high‐SFA meal. Black symbols; baseline measurements, White symbols; after DI‐period. Values are mean ± SEM.

As reported previously, fasting and postprandial NEFA and glycerol concentrations were similar before and after DI‐period (Fig. 2A, C) (Vink et al. 2017a). Furthermore, net fasting and postprandial adipose tissue NEFA and glycerol adipose tissue release were not altered by the DI‐period (Fig. 2B and D). Fasting and postprandial plasma TAG concentrations significantly decreased after the DI‐period (*P *=* *0.007 and *P *=* *0.001, respectively) (Fig. 2E) (Vink et al. 2017a). Using a stable isotope tracer methodology, we were able to determine adipose tissue meal‐derived TAG extraction (TAG labeled with [U‐^13^C]‐palmitate) (Bickerton et al. 2007). At baseline and after the DI‐period, the [U‐^13^C]‐palmitate tracer appeared in the plasma TAG fraction 60 min after meal ingestion (Fig. 2F). The net flux of [U‐^13^C]‐palmitate in TAG, which reflects LPL‐mediated hydrolysis of meal‐derived TAG, was not affected by the DI‐period (Fig. 2G) (Vink et al. 2017a).

### Plasma ANGPTL4 and in vivo markers for adipose tissue lipid metabolism

Next, we examined whether the fasting and postprandial plasma ANGPTL4 concentrations were associated with in vivo markers of adipose tissue lipid metabolism before and after weight loss. First, we examined whether postprandial (changes in) plasma ANGPTL4 concentrations after a high‐SFA meal were associated with in vivo adipose tissue LPL activity. There was no relationship between postprandial plasma ANGPTL4 concentrations and the SFA meal‐derived TAG flux, a marker for LPL activity, before and after DI‐period (before: AUC_0–5 h_: *r *=* *0.163; *P *=* *0.546; after DI‐period: AUC_0–5 h_: *r *=* *−0.192; *P *=* *0.477). Moreover, weight loss‐induced changes in ANGPTL4 concentrations were not related to the changes in SFA meal‐derived TAG fluxes.

**Figure 3:**
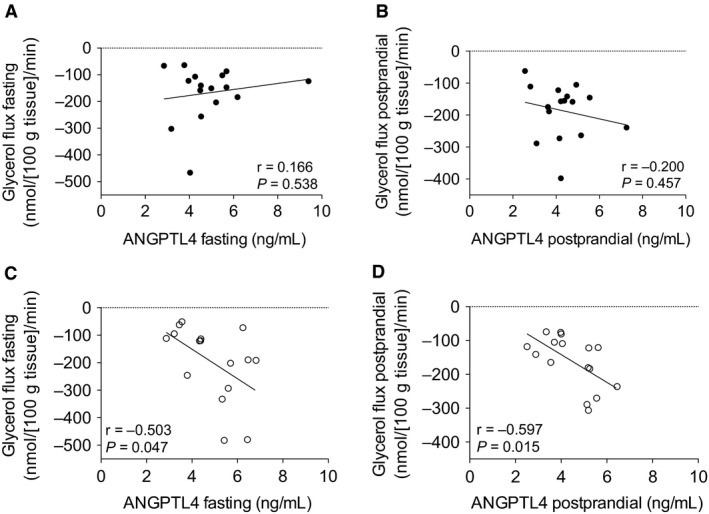
Correlation between plasma ANGPTL4 concentrations and adipose tissue glycerol flux across adipose tissue during fasting conditions at baseline (A) and after DI‐period (C); correlation between postprandial plasma ANGPTL4 concentrations and postprandial adipose tissue glycerol flux across adipose tissue at baseline (B) and after DI‐period (D). Black symbols; baseline measurements, White symbols; after DI‐period. Correlations were analyzed by Pearson's R, *P*‐values <0.05 were considered statistically significant.

Secondly, ANGPTL4 has been reported to stimulate fasting‐induced intracellular lipolysis in adipocytes (Gray et al. 2012). Therefore, we correlated fasting and postprandial plasma ANGPTL4 concentrations with adipose tissue NEFA and adipose tissue glycerol flux under fasting and postprandial conditions. Before the DI‐period, there was no relationship between circulating ANGPTL4 and adipose tissue NEFA release (data not shown) or adipose tissue glycerol release (Fig. 3A and B). Moreover, the DI‐period‐induced plasma ANGPTL4 changes and DI‐period‐induced adipose tissue NEFA release changes were not associated. However, after weight loss, plasma ANGPTL4 was positively associated with fasting and postprandial adipose tissue NEFA release (fasting: *r *=* *0.520; *P *=* *0.039 and AUC_0–5 h_: *r *=* *0.690; *P *=* *0.003). Comparable results were observed for the relation between plasma ANGPTL4 and adipose tissue glycerol release after the DI‐period (fasting: *r *=* *0.503; *P *=* *0.047 (Fig. 3C) and AUC_0–5 h_: *r *=* *0.597; *P *=* *0.015 (Fig. 3D). This indicates that higher plasma ANGPTL4 concentrations are associated with higher adipose tissue NEFA and glycerol release after weight loss.

### ANGPTL4 mRNA levels

To obtain further insight into the relationship between ANGPTL4 and adipose tissue lipid metabolism, a targeted microarray analysis was performed. We selected genes directly involved in adipose tissue FA uptake, lipid synthesis, lipid droplet formation, and lipolysis (Table S1). In accordance with unchanged adipose tissue FA metabolism after weight loss, this targeted microarray approach revealed no significant changes in mRNA levels related to adipose tissue lipid metabolism after the DI‐period as compared to baseline values (Vink et al. 2017a). mRNA levels of LPL and ANGPTL4 were also comparable before and after the DI‐period (Table S1) (Vink et al. 2017a). No associations were found between adipose tissue ANGPTL4 mRNA levels and markers of lipolysis (including *LPL*,* LIPE*,* MGLL,* and *PNPLA2*) before and after the DI‐period. In addition, no associations were found between adipose tissue ANGPTL4 mRNA levels and in vivo adipose tissue LPL activity and the net adipose tissue NEFA and glycerol release at both time points (data not shown).

## Discussion

This study demonstrated that fasting and postprandial plasma ANGPTL4 concentrations were unaffected by weight loss in overweight and obese humans. Furthermore, we found that ANGPTL4 was released from adipose tissue into the circulation after a high‐SFA meal in humans, and this release tended to increase after weight loss. There was no association between plasma ANGPTL4 and in vivo adipose tissue LPL activity, neither before nor after weight loss. However, after weight loss, plasma ANGPTL4 was positively associated with fasting and postprandial adipose tissue NEFA and glycerol release, reflecting adipose tissue lipolysis.

Circulating ANGPTL4 concentrations have been shown to increase after prolonged fasting and short‐term and chronic caloric restriction (Kersten et al. 2009; Jonker et al. 2013). Importantly, in these studies, participants were still in a negative energy balance when plasma ANGPTL4 was measured. The present study demonstrated that weight loss *per se* does not affect fasting and postprandial plasma ANGPTL4 concentrations and adipose tissue ANGPTL4 gene expression. This is contradictory to a study by Cullberg et al. (2013). They showed that after 8 weeks of VLCD and 4 weeks of weight maintenance diet, plasma ANGPTL4 concentrations increased in obese male and female participants, while adipose tissue ANGPTL4 mRNA expression was decreased (Cullberg et al. 2013). However, their study population was younger, had a different sex distribution, and their participants lost on average 12%, while the average weight loss was 9% in the present study. These factors might partly explain the observed differences. We observed no differences between diets (VLCD and LCD) with respect to changes in circulating ANGPTL4 concentrations.

Here, we provide the first direct evidence that adipose tissue contributes to plasma ANGPTL4 in vivo in humans. Previous research showed that ANGPTL4 is not only induced under fasting conditions but that fatty acids, as infused during a hyperinsulinemic‐euglycemic clamp, can also increase plasma ANGPTL4 concentrations, likely in a peroxisome proliferator‐activated receptors (PPAR)‐mediated manner (Jonker et al. 2013). However, Dijk et al. recently showed a 38% decrease of ANGPTL4 mRNA levels in subcutaneous adipose tissue after a high‐fat mixed meal (Dijk W et al. 2018). Importantly, our adipose tissues biopsies were only taken under fasting conditions; therefore, it remains unclear how our high‐fat mixed meal, which had about one‐third lower caloric content, affected ANGPLT4 mRNA levels. Alternatively, it is tempting to speculate that dissociation of ANGPTL4 from LPL in the vascular area may explain postprandial adipose tissue ANGPLT4 release. At the endothelial membrane, ANGPTL4 inhibits LPL activity via transient binding to LPL (Sukonina et al. 2006). During postprandial conditions, as adipose tissue LPL activity increases, it can be speculated that ANGPTL4 dissociates with LPL at the endothelium. If ANGPTL4 is not taken up again by the underlying tissue (i.e., adipose tissue), this could result in significant ANGPTL4 release into the circulation. After weight loss, the ANGPTL4 secretion from adipose tissue tended to be elevated, although plasma ANGPTL4 concentrations were not affected. A reduced adipose tissue mass together with a higher net adipose tissue ANGPTL4 release per unit adipose tissue might explain comparable circulating ANGPTL4 concentrations before and after the DI‐period. The relative adipose tissue‐derived contribution to circulating concentrations is difficult to estimate as information on ANGPTL4 half‐life is lacking. The fact that plasma ANGPTL4 concentrations decline after the high‐fat meal, despite the increase in ANGPTL4 release from adipose tissue, might indicate that adipose tissue is not the major organ regulating postprandial plasma ANGPTL4 concentrations. Furthermore, we could not detect significant adipose tissue release under fasting conditions before and after weight loss, which suggests that release from other tissues than adipose tissue, such as liver or intestines, determines plasma ANGPTL4 concentrations in humans.

ANGPTL4 plays an important role in tissue‐specific LPL inhibition (Dijk and Kersten 2014). However, in this study we could not show a relationship between circulating ANGPTL4 and in vivo adipose tissue LPL activity before and after weight loss in humans. Recently, we have also shown that plasma ANGPTL4 was not related to skeletal muscle LPL activity in overweight and obese humans with impaired glucose homeostasis (van der Kolk et al. 2016a). These results suggest that circulating ANGPTL4 is not a regulator of LPL activity during postprandial conditions in key metabolic organs. However, ANGPTL4 might be a local regulator of LPL activity via autocrine and paracrine actions and plasma ANGPTL4 might not reflect local effects on adipose tissue LPL activity. Indeed, tissue‐specific LPL activity regulation by ANGPTL4 has been shown to take place in the subendothelial space rather than at the endothelium (Sukonina et al. 2006; Makoveichuk et al. 2013). It has been proposed that newly synthesized and locally produced ANGPTL4 might have more pronounced effects on LPL activity than circulating ANGPTL4 (Nilsson et al. 2012). Recently, Dijk et al. (2016) has shown that locally produced ANGPTL4 promotes intracellular degradation of LPL in murine adipocytes. Additionally, more factors are involved in functional ANGPTL4 activity regarding LPL inhibition. It has been suggested that the locally produced glycosylphosphatidylinositol‐anchored high‐density lipoprotein‐binding protein 1 (GPIHBP1) might compete with ANGPTL4 for LPL binding at either the interstitial space or capillary lumen (Dijk and Kersten 2016). This indicates that there is a complex regulation of in vivo LPL activity with many factors involved.

Next to LPL inhibition, it has been suggested that ANGPTL4 stimulates intracellular lipolysis in adipocytes in the fasted state, thereby favoring the use of NEFAs as fuel by other tissues (Dijk and Kersten 2016). In the present study, after weight loss plasma ANGPTL4 was positively associated with estimates of adipose tissue lipolysis (i.e., adipose tissue NEFA and glycerol release) under fasting and postprandial conditions. It is tempting to speculate that weight loss might improve posttranslational ANGPTL4 signaling in adipose tissue, which might be reflected in the observed association. Up to now, plasma ANGPTL4 has been positively associated with plasma NEFA concentrations during an oral glucose drink, another estimate of adipose tissue lipolysis (Staiger et al. 2009). In transgenic ANGPTL4 mice models, an altered release of NEFA and glycerol from adipose tissue explants was observed (Sanderson et al. 2009). In ANGPTL4 knockout mice, a lack of NEFA increase during the fasted state was observed (Sanderson et al. 2009). Furthermore, in vitro studies have shown that ANGPTL4 markedly induced glycerol release from 3T3‐L1 adipocytes (Sanderson et al. 2009) and that ANGPTL4 is a glucocorticoid‐responsive mediator which stimulates fasting‐induced intracellular lipolysis by enhancing cAMP signaling in adipocytes (Gray et al. 2012). Finally, it has been shown that adipose tissue ANGPTL4 mRNA expression was positively correlated with HSL and *ABHD5/CGI‐58* gene expression in the Finnish Twin cohort (Robciuc MR et al. 2012). However, in the present study adipose tissue expression of ANGPTL4 was not associated with markers of lipolysis in overweight and obese participants. Together, these results suggest that plasma ANGPTL4 might play a role in adipose tissue lipolysis and possibly may play a role in lipid partitioning after weight loss.

The present study had some limitations. First, we have measured full‐length ANGPTL4 and the C‐terminal truncated fragment, but not the N‐terminal truncated fragment of ANGPTL4 (Jonker et al. 2013). However, both the full‐length and the N‐terminal fragment ANGPTL4 may influence plasma lipid concentration (Yin et al. 2009). Therefore, it remains to be established whether the results from the ANGPTL4 ELISA provide an accurate reflection of functional ANGPTL4. Secondly, we cannot exclude the possibility that the participants were still in a negative energy balance after the 4‐week WS period (Vink et al. 2017b). This might have partly impacted the observed ANGPTL4 effects after weight loss. Moreover, an intervention with more pronounced effects of weight loss on LPL activity, might also show a different effect on ANGPTL4 expression and plasma concentrations (Cullberg et al. 2013). Finally, it has been shown that there may be sex differences in fatty acid handling in humans (Varlamov et al. 2014). However, the present study was not powered to analyze sex‐specific differences in fatty acid metabolism.

In conclusion, we provide in vivo evidence that ANGPTL4 is secreted from human abdominal subcutaneous adipose tissue after a high‐SFA meal, which tended to be elevated after weight loss. Furthermore, our data indicate that plasma ANGPTL4 concentrations do not relate to in vivo adipose tissue LPL activity. However, after weight loss, there was a positive association between plasma ANGPTL4 and fasting and postprandial abdominal subcutaneous adipose tissue NEFA and glycerol release, partly reflecting adipose tissue lipolysis. Therefore, the role of ANGPTL4 in human adipose tissue lipolysis needs to be investigated in more detail in future research.

## Conflict of Interest

The authors declare that there is no duality of interest associated with this manuscript.

## Data Accessibility

## Supporting information




**Table S1.** List of selected genes important for AT FA handling in humans from the microarray analysis and their log_2_ expression values.

## References

[phy213735-bib-0001] Alex, S. , L. Lichtenstein , W. Dijk , R. P. Mensink , N. S. Tan , and S. Kersten . 2013 ANGPTL4 is produced by entero‐endocrine cells in the human intestinal tract. Histochem. Cell Biol. 141:383–391. 24141811 10.1007/s00418-013-1157-y

[phy213735-bib-0002] Bickerton, A. S. T. , R. Roberts , B. A. Fielding , L. Hodson , E. E. Blaak , A. J. M. Wagenmakers , et al. 2007 Preferential uptake of dietary fatty acids in adipose tissue and muscle in the postprandial period. Diabetes 56:168–176. 17192479 10.2337/db06-0822

[phy213735-bib-0003] Catoire, M. , S. Alex , N. Paraskevopulos , F. Mattijssen , I. Evers‐van Gogh , G. Schaart , et al. 2014 Fatty acid‐inducible ANGPTL4 governs lipid metabolic response to exercise. Proc. Natl Acad. Sci. USA 111:E1043–E1052. 24591600 10.1073/pnas.1400889111 PMC3964070

[phy213735-bib-0004] Cullberg, K. B. , T. Christiansen , S. K. Paulsen , J. M. Bruun , S. B. Pedersen , and B. Richelsen . 2013 Effect of weight loss and exercise on angiogenic factors in the circulation and in adipose tissue in obese subjects. Obesity. 21:454–460. 23401397 10.1002/oby.20060

[phy213735-bib-0005] Dijk, W. , and S. Kersten . 2014 Regulation of lipoprotein lipase by Angptl4. Trends Endocrinol. Metab. 25:146–155. 24397894 10.1016/j.tem.2013.12.005

[phy213735-bib-0006] Dijk, W. , and S. Kersten . 2016 Regulation of lipid metabolism by angiopoietin‐like proteins. Curr. Opin. Lipidol. 27:249–256. 27023631 10.1097/MOL.0000000000000290

[phy213735-bib-0007] Dijk, W. , S. Schutte , E. O. Aarts , I. M. C. Janssen , L. Afman , and S. Kersten . Regulation of angiopoietin‐like 4 and lipoprotein lipase in human adipose tissue. J. Clin. Lipidol. 2018; https://doi.org/10.1016/j.jacl.2018.02.006. 10.1016/j.jacl.2018.02.006 29555209

[phy213735-bib-0008] Dijk, W. , A. P. Beigneux , M. Larsson , A. Bensadoun , S. G. Young , and S. Kersten . 2016 Angiopoietin‐like 4 promotes intracellular degradation of lipoprotein lipase in adipocytes. J. Lipid Res. 57:1670–1683. 27034464 10.1194/jlr.M067363 PMC5003152

[phy213735-bib-0009] Farese, R. V. , T. J. Yost , and R. H. Eckel . 1991 Tissue‐specific regulation of lipoprotein‐lipase activity by insulin glucose in normal‐weight humans. Metab., Clin. Exp. 40:214–216. 1988780 10.1016/0026-0495(91)90178-y

[phy213735-bib-0010] Goldberg, I. J. , R. H. Eckel , and N. A. Abumrad . 2009 Regulation of fatty acid uptake into tissues: lipoprotein lipase‐ and CD36‐mediated pathways. J. Lipid Res. 50(Suppl):S86–S90. 19033209 10.1194/jlr.R800085-JLR200 PMC2674753

[phy213735-bib-0011] Gray, N. E. , L. N. Lam , K. Yang , A. Y. Zhou , S. Koliwad , and J.‐C. Wang . 2012 Angiopoietin‐like 4 (Angptl4) protein is a physiological mediator of intracellular lipolysis in murine adipocytes. J. Biol. Chem. 287:8444–8456. 22267746 10.1074/jbc.M111.294124 PMC3318686

[phy213735-bib-0012] Jocken, J. W. E. , G. H. Goossens , A. M. J. van Hees , K. N. Frayn , M. van Baak , J. Stegen , et al. 2008 Effect of beta‐adrenergic stimulation on whole‐body and abdominal subcutaneous adipose tissue lipolysis in lean and obese men. Diabetologia 51:320–327. 18060661 10.1007/s00125-007-0866-y PMC2170457

[phy213735-bib-0013] Jonker, J. T. , J. W. Smit , S. Hammer , M. Snel , R. W. van der Meer , H. J. Lamb , et al. 2013 Dietary modulation of plasma angiopoietin‐like protein 4 concentrations in healthy volunteers and in patients with type 2 diabetes. Am. J. Clin. Nutr. 97:255–260. 23283507 10.3945/ajcn.112.043687

[phy213735-bib-0014] Kersten, S. 2014 Physiological regulation of lipoprotein lipase. Biochim. Biophys. Acta 1841:919–933. 24721265 10.1016/j.bbalip.2014.03.013

[phy213735-bib-0015] Kersten, S. , S. Mandard , N. S. Tan , P. Escher , D. Metzger , P. Chambon , et al. 2000 Characterization of the fasting‐induced adipose factor FIAF, a novel peroxisome proliferator‐activated receptor target gene. J. Biol. Chem. 275:28488–28493. 10862772 10.1074/jbc.M004029200

[phy213735-bib-0016] Kersten, S. , L. Lichtenstein , E. Steenbergen , K. Mudde , H. F. J. Hendriks , M. K. Hesselink , et al. 2009 Caloric restriction and exercise increase plasma ANGPTL4 levels in humans via elevated free fatty acids. Arterioscler. Thromb. Vasc. Biol. 29:969–974. 19342599 10.1161/ATVBAHA.108.182147

[phy213735-bib-0017] van der Kolk, B. W. , G. H. Goossens , J. W. Jocken , S. Kersten , and E. E. Blaak . 2016a Angiopoietin‐like protein 4 and postprandial skeletal muscle lipid metabolism in overweight and obese prediabetics. J. Clin. Endocrinol. Metab. 101:2332–2339. 27011113 10.1210/jc.2015-4285

[phy213735-bib-0018] van der Kolk, B. W. , G. H. Goossens , J. W. Jocken , and E. E. Blaak . 2016b Altered skeletal muscle fatty acid handling is associated with the degree of insulin resistance in overweight and obese humans. Diabetologia 59:2686–2696. 27627982 10.1007/s00125-016-4104-3 PMC6518064

[phy213735-bib-0019] Köster, A. , Y. B. Chao , M. Mosior , A. Ford , P. A. Gonzalez‐DeWhitt , J. E. Hale , et al. 2005 Transgenic angiopoietin‐like (angptl)4 overexpression and targeted disruption of angptl4 and angptl3: regulation of triglyceride metabolism. Endocrinology 146:4943–4950. 16081640 10.1210/en.2005-0476

[phy213735-bib-0020] Kroupa, O. , E. Vorrsj , R. Stienstra , F. Mattijssen , S. K. Nilsson , V. Sukonina , et al. 2012 Linking nutritional regulation of Angptl4, Gpihbp1, and Lmf1 to lipoprotein lipase activity in rodent adipose tissue. BMC Physiol. 12:1–1. 23176178 10.1186/1472-6793-12-13 PMC3562520

[phy213735-bib-0021] Makoveichuk, E. , E. Vorrsjö , T. Olivecrona , and G. Olivecrona . 2013 Inactivation of lipoprotein lipase in 3T3‐L1 adipocytes by angiopoietin‐like protein 4 requires that both proteins have reached the cell surface. Biochim. Biophys. Acta 441:941–946. 10.1016/j.bbrc.2013.11.013 24220340

[phy213735-bib-0022] Miles, J. M. , Y. S. Park , D. Walewicz , C. Russell‐Lopez , S. Windsor , W. L. Isley , et al. 2004 Systemic and forearm triglyceride metabolism: fate of lipoprotein lipase‐generated glycerol and free fatty acids. Diabetes 53:521–527. 14988233 10.2337/diabetes.53.3.521

[phy213735-bib-0023] Nilsson, S. K. , F. Anderson , M. Ericsson , M. Larsson , E. Makoveichuk , A. Lookene , et al. 2012 Triacylglycerol‐rich lipoproteins protect lipoprotein lipase from inactivation by ANGPTL3 and ANGPTL4. Biochim. Biophys. Acta 1821:1370–1378. 22732211 10.1016/j.bbalip.2012.06.003

[phy213735-bib-0024] Norheim, F. , M. Hjorth , T. M. Langleite , S. Lee , T. Holen , C. Bindesbøll , et al. Regulation of angiopoietin‐like protein 4 production during and after exercise. Physiol. Rep. 2014;2:1–12. https://doi.org/10.14814/phy2.12109 10.14814/phy2.12109 PMC4246580 25138789

[phy213735-bib-0025] Robciuc, M. R. , P. Skrobuk , A. Anisimov , V. M. Olkkonen , K. Alitalo , R. H. Eckel , et al. Angiopoietin‐like 4 mediates PPAR delta effect on lipoprotein lipase‐dependent fatty acid uptake but not on beta‐oxidation in myotubes. PLoS One. 2012;7:e46212. 23056264 10.1371/journal.pone.0046212 PMC3464237

[phy213735-bib-0026] Sanderson, L. M. , T. Degenhardt , A. Koppen , E. Kalkhoven , B. Desvergne , M. Müller , et al. 2009 Peroxisome proliferator‐activated receptor beta/delta (PPARbeta/delta) but not PPARalpha serves as a plasma free fatty acid sensor in liver. Mol. Cell. Biol. 29:6257–6267. 19805517 10.1128/MCB.00370-09 PMC2786701

[phy213735-bib-0027] Staiger, H. , C. Haas , J. Machann , R. Werner , M. Weisser , F. Schick , et al. 2009 Muscle‐derived angiopoietin‐like protein 4 is induced by fatty acids via peroxisome proliferator‐activated receptor (PPAR)‐delta and is of metabolic relevance in humans. Diabetes 58:579–589. 19074989 10.2337/db07-1438 PMC2646056

[phy213735-bib-0028] Stinkens, R. , G. H. Goossens , J. W. E. Jocken , and E. E. Blaak . 2015 Targeting fatty acid metabolism to improve glucose metabolism. Obes. Rev. 16:715–757. 26179344 10.1111/obr.12298

[phy213735-bib-0029] Sukonina, V. , A. Lookene , T. Olivecrona , and G. Olivecrona . 2006 Angiopoietin‐like protein 4 converts lipoprotein lipase to inactive monomers and modulates lipase activity in adipose tissue. Proc. Natl Acad. Sci. USA 103:17450–17455. 17088546 10.1073/pnas.0604026103 PMC1859949

[phy213735-bib-0030] Varlamov, O. , C. L. Bethea , and C. T. Roberts . 2014 Sex‐specific differences in lipid and glucose metabolism. Front. Endocrinol. (Lausanne). 5:241. 25646091 10.3389/fendo.2014.00241 PMC4298229

[phy213735-bib-0031] Vink, R. G. , N. J. T. Roumans , L. A. J. Arkenbosch , E. C. M. Mariman , and M. A. van Baak . 2016 The effect of rate of weight loss on long‐term weight regain in adults with overweight and obesity. Obesity. 24:321–327. 26813524 10.1002/oby.21346

[phy213735-bib-0032] Vink, R. G. , N. J. Roumans , B. W. van der Kolk , P. Fazelzadeh , M. V. Boekschoten , E. C. Mariman , et al. 2017a Adipose tissue meal‐derived fatty acid uptake before and after diet‐induced weight loss in adults with overweight and obesity. Obesity. 25:1391–1399. 28639346 10.1002/oby.21903

[phy213735-bib-0033] Vink, R. G. , N. J. Roumans , P. Fazelzadeh , S. H. K. Tareen , M. V. Boekschoten , M. A. van Baak , et al. 2017b Adipose tissue gene expression is differentially regulated with different rates of weight loss in overweight and obese humans. Int. J. Obes. (Lond). 41:309–316. 27840413 10.1038/ijo.2016.201

[phy213735-bib-0034] Yin, W. , S. Romeo , S. Chang , N. V. Grishin , H. H. Hobbs , and J. C. Cohen . 2009 Genetic variation in ANGPTL4 provides insights into protein processing and function. J. Biol. Chem. 284:13213–13222. 19270337 10.1074/jbc.M900553200 PMC2676053

