# Supplementary material for: Effect of diet‐induced weight loss on angiopoietin‐like protein 4 and adipose tissue lipid metabolism in overweight and obese humans
Source: Physiol Rep. 2018 Jul 12;6(13):e13735. doi: 10.14814/phy2.13735 (PMC6041698; doi:10.14814/phy2.13735)
Supplement: Supplementary file 1 — Table S1. List of selected genes important for AT FA handling in humans from the microarray analysis and their log2 expression values. [file PHY2-6-e13735-s001.docx]

**Supplementary Table 1. List of selected genes important for AT FA handling in humans from the microarray analysis and their log_2_ expression values.**

|  | | T1  (Log_2_ expression) | T3  (Log_2_ expression) |
| --- | --- | --- | --- |
| FA uptake | |  |  |
| *LPL* | | 11.18 ± 0.27 | 11.12 ± 0.17 |
| *ANGPTL4* | | 7.52 ± 0.40 | 7.40 ± 0.33 |
| *PPARy* | | 9.37 ± 0.23 | 9.38 ± 0.25 |
| *SREBF1* | | 6.65 ± 0.49 | 6.76 ± 0.48 |
| FA synthesis | |  |  |
| *FASN* | | 9.37 ± 0.57 | 9.10 ± 0.50 |
| *ACACA* | | 7.07 ± 0.40 | 6.94 ± 0.35 |
| *SCD* | | 11.02 ± 0.87 | 10.67 ± 0.65 |
| TAG synthesis | |  |  |
| *GPAM* | | 11.29 ± 0.53 | 11.10 ± 0.39 |
| *AGPAT9* | | 4.97 ± 0.59 | 5.08 ± 0.61 |
| *DGAT1* | | 7.86 ± 0.28 | 7.84 ± 0.21 |
| *DGAT2* | | 9.75 ± 0.54 | 9.48 ± 0.62 |
| *AQP7* | | 10.24 ± 0.25 | 10.31± 0.20 |
| Lipid droplet formation | |  |  |
| *FITM2* | | 6.80 ± 0.22 | 7.02 ± 0.26 * |
| *CIDEC* | | 10.23 ± 0.30 | 10.33 ± 0.21 |
| *PLIN1* | | 11.58 ± 0.20 | 11.58 ± 0.21 |
| Intracellular lipolysis | |  |  |
| PNPLA2 | | 10.40 ± 0.31 | 10.30 ± 0.16 |
| *LIPE* | | 9.41 ± 0.24 | 9.25 ± 0.24 |
| *CGI-58* | | 8.28 ± 0.38 | 8.33 ± 0.38 |
| *MGLL* | | 9.64 ± 0.28 | 9.48 ± 0.25 |
| *ADRB2* | | 7.17 ± 0.28 | 7.28 ± 0.35 |
| *CIDEA* | | 7.66 ± 0.60 | 7.86 ± 0.67 |
| *G0/S2* | | 11.12 ± 0.19 | 11.13 ± 0.24 |
| Genes that did not meet filter critera | | |  |
| *NPRA* |  | |  |
| *FABP4* |  | |  |
| *CD36* |  | |  |
| *FATP* |  | |  |
| *ANP* |  | |  |
| *BNP* |  | |  |

Values are mean ± SD. * for difference between start study and after DI-period, student’s *t*-test for paired samples
